# Retrieval-augmented generation for generative artificial intelligence in health care

**DOI:** 10.1038/s44401-024-00004-1

**Published:** 2025-01-25

**Authors:** Rui Yang, Yilin Ning, Emilia Keppo, Mingxuan Liu, Chuan Hong, Danielle S. Bitterman, Jasmine Chiat Ling Ong, Daniel Shu Wei Ting, Nan Liu

**Affiliations:** 1 Center for Quantitative Medicine, Duke-NUS Medical School, Singapore, Singapore; 2 Faculty of Arts and Science, University of Toronto, Toronto, ON Canada; 3 Department of Biostatistics and Bioinformatics, Duke University, Durham, NC USA; 4 Artificial Intelligence in Medicine Program, Mass General Brigham, Harvard Medical School, Boston, MA USA; 5 Division of Pharmacy, Singapore General Hospital, Singapore, Singapore; 6 Singapore Eye Research Institute, Singapore National Eye Center, Singapore, Singapore; 7 Byers Eye Institute, Stanford University, Stanford, CA USA; 8 Program in Health Services and Systems Research, Duke-NUS Medical School, Singapore, Singapore; 9 NUS Artificial Intelligence Institute, National University of Singapore, Singapore, Singapore

**Keywords:** Health care, Medical research

## Abstract

Generative artificial intelligence has brought disruptive innovations in health care but faces certain challenges. Retrieval-augmented generation (RAG) enables models to generate more reliable content by leveraging the retrieval of external knowledge. In this perspective, we analyze the possible contributions that RAG could bring to health care in equity, reliability, and personalization. Additionally, we discuss the current limitations and challenges of implementing RAG in medical scenarios.

## Introduction

Generative artificial intelligence (AI) has recently attracted widespread attention across various fields, including the GPT^1,2^ and LLaMA^3,4^ series for text generation, DALL-E^5^ for image generation, as well as Sora^6^ for video generation. In health care systems, generative AI holds promise for applications in consulting, diagnosis, treatment, management, and education^7,8^. Additionally, the utilization of generative AI could enhance the quality of health services for patients while alleviating the workload for clinicians^8–10^.

Despite this, it is crucial to acknowledge the inherent limitations of generative AI models, which include susceptibility to biases from pre-training data^11^, lack of transparency, the potential to generate incorrect content, and difficulty in maintaining up-to-date knowledge, among others^7^. For instance, large language models (LLMs) were shown to generate biased responses by adopting outdated race-based equations to estimate renal function^12^. In the process of image generation, biases related to gender, skin tone, and geo-cultural factors have been observed^13^. Similarly, for downstream tasks such as question answering, LLM-generated content is often factually inconsistent and lacks evidence for verification^14^. Moreover, due to their static knowledge and inability to access external data, generative AI models are unable to provide up-to-date clinical advice for physicians or effective personalized health management for patients^15^.

**Fig. 1:**
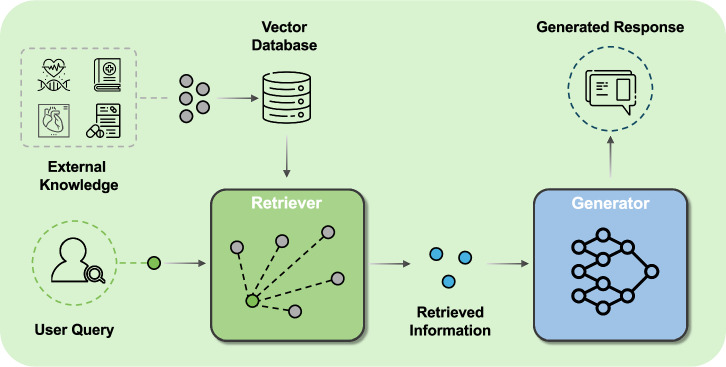
A typical retrieval-augmented generation framework. External data is first encoded into vectors and stored in the vector database (where vectors are mathematical representations of various types of data in a high-dimensional space). In the retrieval stage, when receiving a user query, the retriever searches for the most relevant information from the vector database. In the generation stage, both the user’s query and the retrieved information are used to prompt the model to generate content.

In tackling these challenges, retrieval-augmented generation (RAG) has been explored as a potential solution^16,17^. By providing models with externally retrieved data, RAG can enhance the reliability of generated content. A typical RAG framework consists of three parts (Fig. 1): indexing, retrieval, and generation. In the indexing stage, external data is split into chunks, encoded into vectors, and stored in a vector database. In the retrieval stage, the user’s query is encoded into a vector representation, and then the most relevant information is retrieved through similarity calculations between the query and the information in the vector database. The retrieval techniques are not limited to dense retrieval but also include sparse and hybrid retrieval approaches, and advanced reranking methods can be employed to improve the relevance of retrieved content. In the generation stage, both the user’s query and the retrieved relevant information are prompted by the model to generate content. Compared to model fine-tuning for a specific task, RAG is generally more computationally efficient and has been shown to improve accuracy for knowledge-intensive tasks^18^, offering a more flexible paradigm for model updates and integration with other AI techniques.

**Fig. 2:**
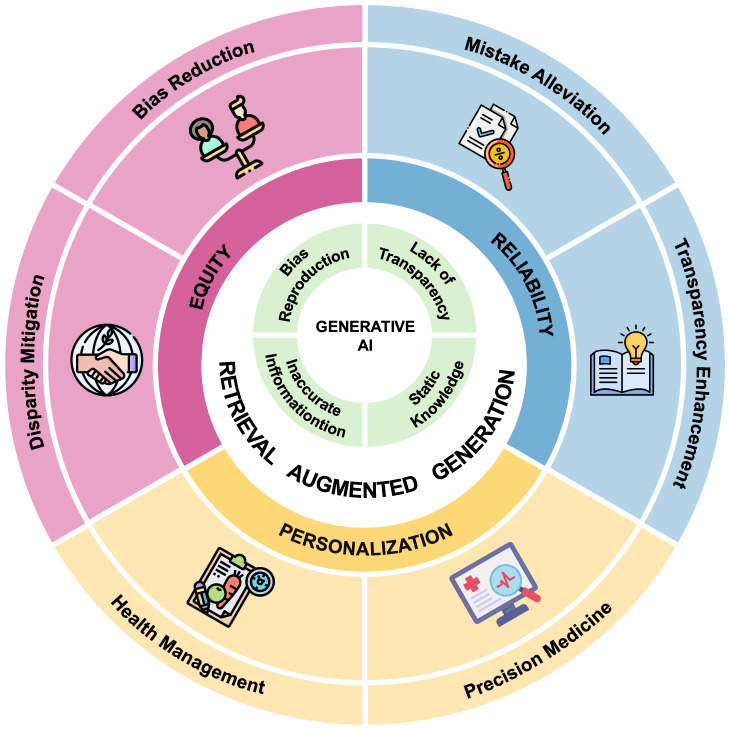
Retrieval-augmented generation could contribute to health care in terms of equity, reliability, and personalization. Generative AI has limitations such as biased reproduction, lack of transparency, inaccurate information, and static knowledge, which hinder its further application in health care. Retrieval-augmented generation holds the potential to alleviate these issues and drive medical innovation from the perspectives of equity, reliability, and personalization.

In this perspective, we discuss the role of RAG in the context of generative AI, particularly its possible applications within health care. We examine RAG’s possible contributions from three aspects: equity, reliability, and personalization (Fig. 2). Additionally, we explore the limitations of RAG in medical application scenarios, emphasizing the need for further research to understand the impact of its implementation within health systems^19^.

## Promoting health equity in generative AI applications

### Bias reduction

The content generated by generative AI models could perpetuate biases inherent in the pre-training data, which are reflected in aspects including demographic characteristics, political ideologies, and sexual orientations^12,13,20^. Such biases can not only lead to unfair diagnoses and treatments but also exacerbate health inequalities for particular populations.

RAG is able to obtain information from external knowledge sources, including medical literature, clinical guidelines, and case reports, to optimize the output of generative AI models^17^. By retrieving information specific to certain subpopulations, the model could analyze a patient’s condition from multiple perspectives, potentially reducing the risk of bias contained in the generated content. For instance, when targeting different gender groups, RAG could retrieve research findings on their specific physiological patterns, common disease spectra, clinical manifestations, as well as related recommendations on clinical practice^21–23^. Similarly, for different ethnic groups, RAG enables access to research reports involving their genetic, environmental, and lifestyle factors, to understand differences in disease incidence rates and unique symptom presentations^24^. Furthermore, for other specific subpopulations (such as different age groups, socioeconomic statuses, etc.), RAG can retrieve tailored medical evidence to help comprehensively understand their unique health needs^25^. Although there remain challenges in ensuring access to high-quality data for underrepresented groups, RAG offers possible solutions to mitigate these issues.

### Disparity mitigation

Health disparities present additional challenges to marginalized groups in accessing medical resources and health services, potentially hindering the achievement of fairness. Although generative AI models are trained on extensive data, the pre-training data itself exhibits imbalances in representing different groups. For example, 92.64% of the pre-training corpus of GPT-3 is derived from English sources, resulting in limited coverage of communities that use other languages^1^. This skewness could make it challenging to meet the medical needs of underrepresented groups.

Collecting data specific to these underrepresented populations and incorporating it into the RAG system holds the potential to mitigate the disparities in health care. Specifically, in low-resource regions, the RAG system might leverage knowledge that integrates local medical research literature, clinical guidelines, and practical experiences to provide more relevant diagnostic and treatment advice to local residents^26^. While some regional guidelines may not be digitized, audio and image recognition technologies could convert this information into digital format, creating region-specific contextual databases^27^. Similarly, by developing high-quality multilingual medical knowledge bases, RAG can play an important role in cross-language information retrieval and knowledge integration, with the potential to eliminate barriers posed by language differences. However, it is worth noting that even the most advanced LLMs currently support only a limited number of mainstream languages, which limits the effectiveness of RAG in multilingual environments, particularly when dealing with languages in low-resource setting^28^. Additionally, RAG systems are able to retrieve pre-collected materials and present them in various formats, such as text, images, and videos, to facilitate patient education. This way allows the explanation of complex medical concepts to patients with diverse educational and cultural backgrounds^29^.

## Generating reliable content

### Mistake alleviation

One significant challenge of generative AI models in health care is their potential to generate incorrect or unfaithful information^7,8^. Although there are already specific models pre-trained on large amounts of medical data, such as Med-PaLM2 and Med-Gemini, the phenomenon of “hallucination” cannot be avoided^29,30^. This issue is extremely sensitive since any false information related to disease diagnosis, treatment plans, or medication guidance will likely cause serious harm to patients^31^.

For example, medication errors are a major category of medical mistakes, resulting in numerous patient fatalities each year^32,33^. During the stage of converting prescription instructions into a standard format, pharmacy technicians may incorrectly record dosage, frequency, or route of administration^32^. Additionally, when patients transfer medications from their original packaging to other containers, it becomes difficult for pharmacists to recognize the medications, which could lead to omission errors^33^. Given that electronic health record recommendations and alerts are often imprecise, and traditional natural language processing methods require extensive human annotation, generative AI offers an attractive solution. However, generative AI models sometimes also generate incorrect drug information, leading to further harm. RAG might help to address some of these issues. By searching various drug information, RAG can automatically parse prescriptions at the data entry stage and generate more accurate medication guidance, thereby reducing medical errors caused by information transmission. Moreover, in the process of drug identification, a multimodal RAG system has the capability to recognize the appearance features of drugs, such as color, shape, and imprints. By matching these characteristics with database information, the RAG system could generate reliable drug information to serve as a reference for pharmacists, thereby improving the efficiency of drug identification. However, it is crucial to emphasize that these applications are still in the early stage of development and require thorough validation before implementation.

### Transparency enhancement

The “black box” nature of generative AI models makes it difficult to explain how specific diagnoses or treatment recommendations are derived. This lack of transparency not only undermines the trust of physicians and patients in the generated content but, more importantly, it may pose serious medical risks and ethical concerns. Although some research has attempted to enhance models’ reasoning abilities and transparency through approaches like chain of thought^34^, multi-agent discussion^35^, and post-hoc attribution^36^, there are still limitations in medical applications^37^.

In comparison, RAG is able to retrieve traceable medical facts from external knowledge bases, promoting the generation of more transparent content; however, this process still requires manual verification^38^. In assisting clinical decision-making, RAG may provide the sources of information upon which the diagnoses are based, including clinical guidelines, medical evidence, and clinical cases. By categorizing queries into simple factual searches or multi-step reasoning processes, RAG can further clarify how different types of information contribute to a given recommendation, enhancing the transparency of its decision-making. Additionally, some research utilizes external medical knowledge graphs (such as the Unified Medical Language System) or self-construed knowledge graphs to enhance the diagnostic capabilities of models^14,39^. Based on a given query, the RAG system first identifies relevant nodes in the knowledge graph, such as diseases, symptoms, or medications, and then retrieves both direct relations and multi-hop paths connecting these nodes. This process allows the RAG system to extract structured, relevant knowledge efficiently and leverage it to provide clear diagnostic explanations^14^.

## Personalizing health care services

### Health management

RAG also shows potential for personalized health care management. Generative AI models lack the ability to incorporate personal information, making it difficult to offer effective health services^8^. For example, they may not be aware of a user’s allergies and recommend allergenic foods. In contrast, the RAG system could integrate health data and lifestyle habits of individuals to build a comprehensive personal profile, which might enable more customized health guidance.

For patients, by connecting their medical records and clinical data while allowing for real-time updates, the RAG system has the capability to provide more precise health management guidance. For instance, for patients with chronic conditions who need to take multiple medications long-term, the system can generate medication reminders according to physicians’ prescriptions, ensuring that patients take their medications correctly and timely, thereby improving medication adherence. For the public, the RAG system can analyze personal health data, lifestyle, environmental factors, and genetic information (if granted access by individual users) to identify potential health risks. In this way, the RAG system provides personalized health recommendations, including diet, exercise, and stress management, effectively promoting disease prevention. For example, for individuals with a high genetic risk of heart disease, the system could recommend specific dietary plans and appropriate exercise regimens to reduce the risk of eventually developing the disease.

### Precision medicine

Precision medicine aims to maximize medical effectiveness and patient benefits by tailoring treatment strategies according to a patient’s genetic profile, environmental influences, lifestyle, and other individual factors^40^. Although current generative AI models have demonstrated potential to assist in clinical decision-making^35,41^, they still face challenges in precision medicine^42^, as they struggle to utilize highly individualized patient data to provide precise treatment recommendations.

RAG might offer unique advantages for advancing precision medicine. By retrieving a patient’s complex clinical and molecular data, the RAG system empowers physicians to develop more accurate and personalized treatment plans tailored to each patient^43^. For example, generative AI models typically provide similar general clinical advice to cancer patients exhibiting similar signs and symptoms. However, in reality, these patients generally have different disease progression and prognoses due to differences in their biomarkers (e.g., DNA, RNA, proteins, metabolites, host cells, and microbiomes)^44^. Although collecting and protecting such sensitive data remains a challenge, RAG could better leverage this information for precision medicine practices. Specifically, the RAG system may be able to comprehensively analyze a patient’s biomarkers, classify them into more granular subgroups, and recommend appropriate personalized treatment plans to physicians based on established clinical guidelines.

## Discussion

RAG may enable better integration of generative AI into health systems and bring more innovative applications in consulting, diagnosis, treatment, management, and education. Despite the potential of RAG systems in health care, they also face significant limitations. First, the retrieval of external knowledge can introduce additional biases, since the sources themselves might contain biases. Second, due to the lack of sufficient high-quality information on underrepresented groups, RAG systems may become less effective in such cases, with the generated content relying more on the knowledge of the models themselves. As a result, minority groups are unlikely to benefit much from existing RAG systems. Third, although RAG systems can enhance transparency by providing evidence, determining which parts of a response are derived from which pieces of retrieved knowledge is difficult without human inspection. Meanwhile, possible knowledge conflicts between retrieved documents or with the model’s internal knowledge highlight the importance of source validation, though effective implementation remains challenging^45^. Fourth, RAG systems face certain privacy risks, as sensitive information stored in retrieval databases can be extracted through designed prompts. Implementing appropriate privacy protection mechanisms is crucial to mitigate the risk of information leakage in generated content, especially when handling sensitive medical information^46^. Therefore, we suggest a multidisciplinary collaboration among clinicians, researchers, stakeholders, and regulators to explore how RAG can be used more equitably, reliably, and effectively to improve existing practices in health care. Such collaboration should focus on addressing practical challenges, including ensuring interoperability with EHR systems, building clinician trust, and providing adequate training for health care professionals to fully harness the potential of RAG^47^.

## Data Availability

No datasets were generated or analysed during the current study.
